# LSPS/Optogenetics to Improve Synaptic Connectivity Mapping: Unmasking the Role of Basket Cell-Mediated Feedforward Inhibition

**DOI:** 10.1523/ENEURO.0142-15.2016

**Published:** 2016-08-08

**Authors:** Julia Brill, Joanna Mattis, Karl Deisseroth, John R. Huguenard

**Affiliations:** 1 Department of Neurology and Neurological Science, Stanford University, Stanford, California 94305; 2 Department of Bioengineering, Stanford University, Stanford, California 94305; 3 Neuroscience Program, Stanford University, Stanford, California 94305; 4 CNC Program, Stanford University, Stanford, California 94305; 5 Department of Psychiatry and Behavioral Sciences, Stanford University, Stanford, California 94305

**Keywords:** connectivity mapping, laser-scanning photostimulation, neocortex, optogenetics, parvalbumin, pyramidal cells

## Abstract

Neocortical pyramidal cells (PYRs) receive synaptic inputs from many types of GABAergic interneurons. Connections between parvalbumin (PV)-positive, fast-spiking interneurons (“PV cells”) and PYRs are characterized by perisomatic synapses and high-amplitude, short-latency IPSCs. Here, we present novel methods to study the functional influence of PV cells on layer 5 PYRs using optogenetics combined with laser-scanning photostimulation (LSPS). First, we examined the strength and spatial distribution of PV-to-PYR inputs. To that end, the fast channelrhodopsin variant AAV5-EF1α-DIO-hChR2(E123T)-eYFP (ChETA) was expressed in PV cells in somatosensory cortex of mice using an adeno-associated virus-based viral construct. Focal blue illumination (100–150 µm half-width) was directed through the microscope objective to excite PV cells along a spatial grid covering layers 2–6, while IPSCs were recorded in layer 5 PYRs. The resulting optogenetic input maps showed evoked PV cell inputs originating from an ∼500-μm-diameter area surrounding the recorded PYR. Evoked IPSCs had the short-latency/high-amplitude characteristic of PV cell inputs. Second, we investigated how PV cell activity modulates PYR output in response to synaptic excitation. We expressed halorhodopsin (eNpHR3.0) in PV cells using the same strategy as for ChETA. Yellow illumination hyperpolarized eNpHR3.0-expressing PV cells, effectively preventing action potential generation and thus decreasing the inhibition of downstream targets. Synaptic input maps onto layer 5 PYRs were acquired using standard glutamate-photolysis LSPS either with or without full-field yellow illumination to silence PV cells. The resulting IPSC input maps selectively lacked short-latency perisomatic inputs, while EPSC input maps showed increased connectivity, particularly from upper layers. This indicates that glutamate uncaging LSPS-based excitatory synaptic maps will consistently underestimate connectivity.

## Significance Statement

Neural computations depend on the interplay of synaptic and intrinsic neuronal properties within complex networks of interconnected neurons. It is of particular interest how individual GABAergic interneuron types modulate network dynamics. Here, we outline novel methods to study connectivity between PV cells, a prominent type of neocortical GABAergic interneuron, and pyramidal cells (PYRs), the main principal neurons of the neocortex. Using optogenetic methods and laser-scanning photostimulation, we map the spatial extent and synaptic characteristics of parvalbumin (PV)-to-PYR connections, and demonstrate a substantial role of PV cells in spatially specific feedforward inhibition of PYRs. These methods can be used to better quantify the role of interneuron types in modulating input to and output of PYRs.

## Introduction

Neocortical interneurons comprise a variety of different types that can be distinguished based on their morphology, marker protein expression patterns, and intrinsic electrophysiological properties (Yuste, 2005; Rudy et al., 2011; Kepecs and Fishell, 2014). They also differ with respect to their role and position within neuronal circuits. For example, parvalbumin (PV)-positive, fast-spiking interneurons (“PV cells”) have relatively short arborizations and synapse onto the cell bodies, proximal dendrites, or axon initial segments of pyramidal cells (PYRs; Marin-Padilla, 1970; Somogyi, 1977). As a result, they provide localized, strong, and fast inhibition; and can veto action potential generation in PYRs (Freund and Katona, 2007), and PYRs and PV cells can dynamically influence each other (Lourenço et al., 2014). Focal activation via glutamate uncaging rapidly recruits powerful perisomatic inhibition (Brill and Huguenard, 2009). At the other extreme, Martinotti cells tend to avoid perisomatic contacts but instead target distal dendrites of PYRs and provide mainly shunting inhibition (Silberberg and Markram, 2007; Murayama et al., 2009). To understand the dynamics of neuronal circuits, it is necessary to tease out the roles of the individual interneuron subtypes. This has been done mostly using anatomical techniques to identify neurons based on morphology and marker protein expression, which allows drawing certain conclusions regarding the connectivity pattern based on their morphology and overlap with arborizations of other cells. Another technique is paired recordings, which reveal the strength and kinetics of the synaptic connections between identified cell types, as well as their connection probability (Thomson and Bannister, 1998; Sotelo, 2003; Bannister and Thomson, 2007; Bock et al., 2011; Packer et al., 2013; van Pelt and van Ooyen, 2013; Qi et al., 2015). These approaches provide valuable insights into cortical circuits that could not easily gained otherwise, but they also have shortcomings: the former technique allows only indirect conclusions regarding synaptic connectivity patterns; and the second technique is tedious and time consuming, and thus not well suited to describe connectivity patterns quantitatively.

A newer method for circuit mapping is the use of laser-scanning photostimulation (LSPS). In LSPS, caged glutamate [i.e., glutamate attached to a chemical moiety that renders it physiologically inert, most commonly 4-methoxy-7-nitroindolinyl (MNI)] is added to the artificial CSF (ACSF) bath. Brief (∼1 ms) and highly focal pulses of UV light photolyze the bond between MNI and glutamate, thus “uncaging” it, and enabling it to act on cellular glutamate receptors. In neurons, only glutamate uncaging onto somata results in action potential generation, while uncaging onto dendrites generally generates subthreshold depolarizations. Thus, focal glutamate uncaging is a method to effectively map the somatic location of the presynaptic inputs of a recorded cell (Callaway and Katz, 1993; Dantzker and Callaway, 2000; Shepherd et al., 2003; Shepherd and Svoboda, 2005; Deleuze and Huguenard, 2006). Further, using two-photon uncaging onto GFP-tagged neurons has the advantage to restrict activation to specific genetically identified neuron types (Fino and Yuste, 2011; Packer and Yuste, 2011).

We developed complementary approaches to combine LSPS and optogenetic circuit mapping to examine PV-to-PYR connectivity more quantitatively: first, we used AAV5-EF1α-DIO-hChR2(E123T)-eYFP (ChETA)-mediated focal excitation of PV cells to map PV-to-PYR inputs analogous to chemical LSPS, but with definite cell-type specificity. This allowed us to unambiguously map PV-to-PYR connections and to identify them conclusively as short-latency inhibitory events. Second, we used LSPS combined with optogenetic silencing of PV cells to record synaptic input maps, and to demonstrate significant layer-specific expansion of excitatory maps under conditions of PV cell silencing. These results show that chemical LSPS with caged glutamate powerfully activates a form of synthetic “feedforward” inhibition that silences neurons, preventing their output, and thus obscuring their contribution to the resultant synaptic connectivity map. This confound must be taken into account in the context of interpreting LSPS-based circuit maps, as the synthetic feedforward inhibition will cause an underestimation of true connectivity. Any changes in the excitability of PV cells, for example in cortical injury (Brill and Huguenard, 2010), would alter the synthetic silencing and further complicate interpretation of altered connectivity maps.

## Materials and Methods

### Animals

All experiments were performed according to protocols approved by Stanford University Institutional Animal Care and Use Committee, and every precaution was taken to minimize stress and the number of animals used in each series of experiments. Parv::cre transgenic mice were obtained from The Jackson Laboratory. Male and female mice aged postnatal day 45–60 were used throughout the experiments.

### Opsin expression

AAV5-EF1α-DIO-eNpHR3.0-EYFP (Gradinaru et al., 2008) and ChETA (Gunaydin et al., 2010) viruses were produced by the University of Carolina at Chapel Hill Vector Core. One microliter of virus (titer 10^12^/μl) was stereotactically injected into bilateral primary somatosensory cortex of 25- to 30-d-old parv::cre mice under isoflurane anesthesia. Injection coordinates (relative to bregma) were as follows: dorsoventral, 0.7; mediolateral, ±1.6; anterioposterior, −0.5.

### Preparation of acute neocortical slices

Acute neocortical slices were prepared 2–4 weeks after virus injection. Mice were deeply anesthetized with 50 mg/kg sodium pentobarbital, and brains were removed and immediately transferred into ice-cold sucrose solution, which contained the following (in mm): 234 sucrose, 11 glucose, 26 NaHCO_3_, 2.5 KCl, 1.25 NaH_2_PO_4_, 10 MgSO_4_, and 0.5 CaCl_2_, equilibrated with 95% O_2_/5% CO_2_. The 300 μm coronal slices were sectioned on a VT 1200S Vibratome (Leica) at 4°C in sucrose solution and transferred to a holding chamber filled with ACSF (in mm: 126 NaCl, 26 NaHCO_3_, 2.5 KCl, 1.25 NaH_3_PO_4_, 2 CaCl_2_, 2 MgCl_2_, 10 glucose, equilibrated with 95% O_2_/5% CO_2_, pH 7.4). After a recovery period of 1 h at 32°C, the holding chamber containing the slices was removed from the water bath and allowed to cool to room temperature.

### Electrophysiological recordings

Slices were transferred to a recording chamber and constantly superfused with oxygenated ACSF at a rate of ∼2 ml/min. Experiments were conducted at room temperature (23–25°C) to avoid excessive evaporation of the relatively small volume (30 ml) of recirculating ACSF containing caged glutamate. All cells recorded were located in the limb area of primary somatosensory cortex. Cortical layers were identified visually from an overview image obtained with a 5× objective and neurons were visualized with a 63× objective using differential contrast optics with an Axioskop 2 FS Microscope (Zeiss). Pyramidal cells were identified based on their large size, tear-shaped morphology, and thick apical dendrite. Opsin-expressing PV cells were identified and targeted based on their enhanced yellow fluorescent protein (eYFP) fluorescence. Recordings were obtained using borosilicate glass electrodes with a tip resistance of 2–4 MΩ. The pipette solution used for voltage-clamp and cell-attached recordings (excitation profiles) contained the following (in mm): 130 Cs-gluconate, 8 CsCl, 10 HEPES, 4 EGTA, and 0.01 QX314, pH 7.3 adjusted with CsOH (290 mOsm). For current-clamp recordings, the following internal solution was used (in mm): 120 K-gluconate, 11 KCl, 1 MgCl_2_, 1 CaCl_2_, 10 HEPES, and 10 EGTA, and pH 7.3 adjusted with KOH (290 mOsm). NMDA receptor-mediated currents were blocked by 50 μm d-AP5 (Ascent Scientific). For recordings of IPSC maps, AMPA and NMDA receptors were blocked, and an internal solution was used that permitted recording IPSCs as inward currents at −60 mV. It contained the following (in mm): 70 K-gluconate, 70 KCl, 2 NaCl, 10 HEPES, 10 EGTA, and 10 EGTA, pH 7.3 adjusted with KOH (290 mOsm). Only recordings in which the series resistance was <25 MΩ and changed <30% during the recording were included in the data analysis, and no series resistance compensation was used. Membrane potentials were corrected for a liquid junction potential of 15 mV, and all voltages given subsequently include liquid junction potential correction. Signals were amplified with a Multiclamp 700A amplifier, sampled at 10 kHz, filtered at 3 kHz, acquired using a Digidata 1320A digitizer, and analyzed using pClamp9 and pClamp10 (all Molecular Devices). Electrical stimulation was performed using a bipolar concentric electrode (CB-X RC75, Frederick Haer).

### Laser-scanning photostimulation/glutamate uncaging

For focal photolysis of caged glutamate, a pulsed 355 nm UV laser beam (DPSS Lasers) was launched into a multimode fiber optic cable and collimated at its output (Oz Optics) then directed via scanning optics into the back aperture of the 5 or 63× microscope objective, so that it could be directed to any point visible through the objective. Scanning was controlled with mirror galvanometers (Model 6210, Cambridge Technology) using a locally developed software program. The beam half-width was ∼130 μm at 5×. Focal photolysis of MNI-caged glutamate (Tocris Bioscience; 100 μm) was triggered by 50 mW UV light pulses (300–800 μs). MNI-caged glutamate was supplied in a 30 ml recirculating bath solution. Typically, the bath solution was exchanged after 3–4 h or sooner if significant rundown of direct responses was detected.

### Optical stimulation

Light produced by a xenon arc lamp (Oligochrome, TILL Photonics) was filtered through a 593 ± 25 nm bandpass filter (FF01-593/40-25, Semrock) and directed into the epifluorescence port of the microscope for full-field illumination. To allow for simultaneous UV illumination (see Fig. 3*A*, diagram), yellow light was reflected using a custom short-pass dichroic mirror [Semrock (similar to SP01-532RU)] that reflected yellow light (593 nm), but passed UV and blue wavelengths (355 and 472 nm, respectively). Alternatively, a blue laser (473 nm; OEM Laser Systems) was scanned onto the slice as described for laser scanning LSPS, using the same Oz Optics multimode optical fiber.

### Synaptic input maps

Using our locally developed software program, we defined areas to be scanned, along with the number of grid points for LSPS within that area (number of rows and columns). Maps spanned all cortical layers with a grid spacing of 90–100 μm. Points were stimulated in a pseudorandom pattern designed to minimize sequential activation of adjacent grid points, with 5 s between stimuli. To isolate EPSC input, maps were recorded at a holding potential of −60 mV, near the chloride equilibrium potential. Direct glutamatergic currents are recorded when glutamate is released onto the recorded cell. These responses consistently have an onset latency of <3 ms and were distinguished from EPSCs on that basis (Brill and Huguenard, 2008). IPSC maps were recorded at −5 mV, close to the reversal potential for AMPA receptor-mediated currents. To construct maps, we determined the cumulative amplitude of PSCs with onset latencies of 3–25 ms (EPSCs) and 2–50 ms (IPSCs) after the stimulus for each sweep. The cumulative amplitude is the sum of the amplitudes of all PSCs recorded within the detection time window and therefore represents a compound measure of event frequency and amplitude. To reduce the confounding effects of polysynaptic activation, NMDA receptors were blocked and a short detection window of 25 ms was used. PSCs were detected using locally written software. We then corrected the cumulative amplitude for the expected spontaneous activity in the equivalent time window. Spontaneous activity was determined for each cell for 1.5 s/sweep during the interstimulus intervals starting 500 ms after each photostimulation. Averaged maps derived from all individual maps were obtained by using exact measurements of the *x* and *y* distances of each stimulation point from the reference point (soma or layer 1/2 border) and then binning these at 100 μm intervals. Smooth contours were derived by linear interpolation between 100 μm bins.

### Immunohistochemistry

Paraformaldehyde fixed and cryoprotected tissue was frozen and cut into 40 µm sections on a HM 400 Cryotome (Microm). Sections were blocked in PBS and 10% normal goat serum for 1 h at room temperature, and incubated in primary antibody overnight at 4°C (mouse anti-parvalbumin; 1:1000; Sigma-Aldrich). Sections were rinsed twice at room temperature for 5 min and then incubated in solution containing fluorescent secondary antibody at room temperature for 1 h (goat anti-mouse IgG Alexa Fluor 568; 2 µg/ml; Invitrogen). After rinsing twice in PBS for 5 min, sections were mounted on SuperfrostPlus slides (Fisher Scientific) and coverslipped using Vectashield Mounting Medium (Vector Laboratories). Images were captured on a LSM 510 Confocal Laser Scanning Microscope (Zeiss).

### Statistical analysis

Error bars reflect SEMs. Statistical significance was calculated using paired or unpaired Student’s *t* tests as appropriate.

## Results

### Opsin expression and functionality

**Figure 1.**
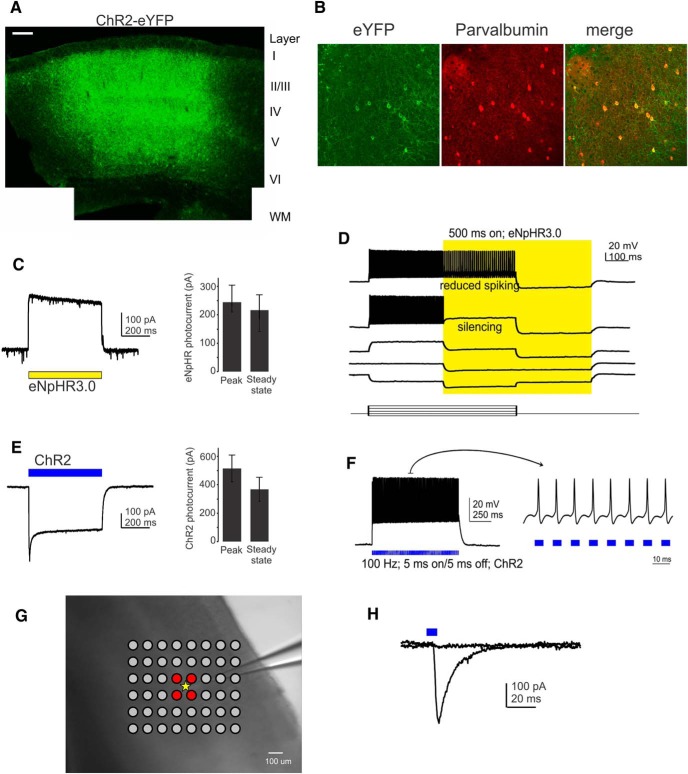
Opsin expression. ***A***, eYFP expresion 3 weeks after virus injection in somatosensory cortex of a P40 mouse. Scale bar, 500 μm. Location of cortical layers and white matter (WM) is indicated. ***B***, Overlapping eYFP and parvalbumin expression demonstrates correct targeting of the viral construct to PV cells. Scale bar, 100 μm. ***C***, Direct eNpHR3.0 current in a PV cell. The yellow bar indicates illumination. ***D***, eNpHR3.0 activation decreases spike output in response to depolarizing current injections (yellow shading, yellow light is turned on). ***E***, Direct ChETA current in a PV cell. The blue bar indicates illumination. ***F***, Current-clamp response showing that PV cells can be driven by ChETA at 100 Hz (5 ms pulse width). The blue bar indicates illumination. ***G***, Stimulation grid surrounding a recorded PV cell (yellow star represents soma location). Red grid points indicate where action potentials were generated in the recorded cell. ***H***, Example traces from a PYR recording showing success and failure in evoking an IPSC in successive trials, demonstrating the all-or-nothing nature of the evoked synaptic response.

Robust eYFP expression was observed 2 weeks after injection (Fig. 1*A*). eYFP expression was observed up to 0.5–1 mm from the injection site. A total of 55.8% of parvalbumin-positive cells within 0.3 mm of the injection site expressed viral protein, as quantified by the presence of eYFP fluorescence, while ∼97% of eYFP-expressing cells were parvalbumin-positive (*n* = 5 slices from three animals; Fig.1*B*). This demonstrated high infection efficiency and specificity.

Photocurrents in response to yellow illumination were readily evoked from PV cells expressing eNpHR3.0 (halorhodopsin); the mean peak photocurrent was 242 ± 63 pA (*n* = 17; Fig. 1*C*), which, given a mean input resistance of 164 ± 36 MΩ (*n* = 13), corresponded to a calculated hyperpolarization of the membrane potential of 25 ± 9 mV. eNpHR3.0-evoked hyperpolarization blocked action potentials over a wide range of depolarizing current injections, and slowed spike frequency at higher injected currents (Fig. 1*D*). In ChETA-expressing PV cells, blue illumination elicited mean peak and steady-state photocurrents of 515 ± 95 and 369 ± 85 pA, respectively (*n* = 5; Fig. 1*E*). Rheobase (i.e., the minimum current needed for suprathreshold depolarization) for PV cells in our experiments was 123 ± 21 pA (range, 50–250 pA; *n* = 11 cells from five animals); thus, the evoked photocurrent would easily drive action potentials. Indeed, repeated 5 ms flashes of blue light delivered at a frequency of 100 Hz reliably evoked spikes in PV cells (*n* = 3; Fig. 1*F*).

### Direct mapping of inputs from ChETA-expressing PV cells onto PYRs

**Figure 2.**
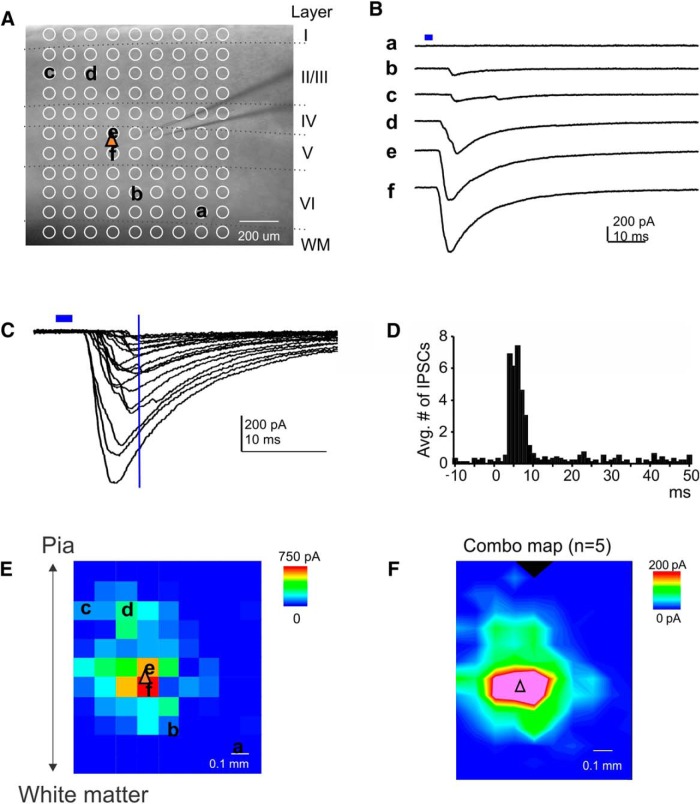
PV-to-PYR connectivity maps. ***A***, Representative grid for recording focal optogenetically evoked IPSCs in a PYR (yellow triangle indicates soma location). Location of cortical layers and white matter is indicated. ***B***, Example traces from the grid in ***A***, at indicated locations. The blue bar indicates the time of stimulus. ***C***, All traces with IPSC responses from the cell in ***A*** and ***B***. The blue horizontal line indicates stimulus time, the blue vertical line indicates 10 ms poststimulus. Note that nearly all IPSCs have a latency of <10 ms. ***D***, Peristimulus time histogram for IPSCs in all recorded PYRs. ***E***, IPSC map from example cell. ***F***, Averaged IPSC map from all recorded PYRs (*n* = 6).

We used focal blue illumination to excite ChETA-expressioning PV cells, and recorded the light-evoked IPSCs in PYRs (Fig. 2*C*). Analogous to LSPS with glutamate uncaging (see above), illumination could be directed quickly and in random order to any position on a user-defined grid within the area visible under the microscope objective. Figure 1*H* shows a representative light-evoked IPSC in a PYR. Traces were recorded at −60 mV using a high-chloride internal solution and ionotropic glutamate receptors were blocked (see Materials and Methods). To map synaptic inputs in a similar way as in LSPS/glutamate uncaging, we needed to determine the direct excitation profile of ChETA-expressing PV cells. Specifically, in chemical LSPS, action potentials are almost exclusively evoked when glutamate is uncaged directly onto the soma or proximal apical dendrite of the cell (Shepherd et al., 2003; Deleuze and Huguenard, 2006), while uncaging on dendrites causes subthreshold depolarizations. However, it is well known that suprathreshold depolarizations can readily be evoked in opsin-expressing axons, and this feature is in fact exploited when characterizing the features or functions of specific synaptic inputs onto a given cell type (Paz et al., 2011; Yizhar et al., 2011; Mattis et al., 2014). Using a relatively low stimulation intensity (∼1 mW) favored somatic over axonal sites of suprathreshold depolarization. Thus, that focal optogenetic stimulation will include the somatic location but may not be entirely limited to it. In the case of ChETA-expressing PV cells, optogenetically evoked suprathreshold depolarizations were restricted to the area surrounding the soma (*n* = 4; Fig. 1*G*), very similar to the situation in LSPS/glutamate uncaging. With that, we were able to map the location of PV cells that projected onto layer 5 PYRs, although a possible contribution from direct axonal activation cannot be excluded (see Discussion).

We typically mapped PV cell inputs onto PYRs from 1 mm^2^ squares using 0.1 mm grid spacing, with the recorded PYR being at the center of the square. Figure 2*A* shows a representative grid and Figure 2*B* depicts example traces recorded at designated grid spots. Most IPSCs were evoked at very short latencies poststimulus (Fig. 2*C*). The poststimulus time histogram in Figure 2*D* illustrates that virtually all evoked IPSCs arose within 10 ms of the light stimulus, which is consistent with the short latency to spike of PV cells (Brill and Huguenard, 2009). Similar to results obtained with two-photon LSPS targeting PV cells (Packer and Yuste, 2011), synaptic inputs originated close to the somata of recorded cells, as seen in the example cell (Fig. 2*E*), and a summary plot (*n* = 5 cells from three animals; Fig. 2*F*). These maps are highly similar to short-latency IPSC input maps and thus confirm that they represent PV-to-PYR inputs (Brill and Huguenard, 2009).

### Mapping PV-to-PYR inputs using PV cell silencing and LSPS

**Figure 3.**
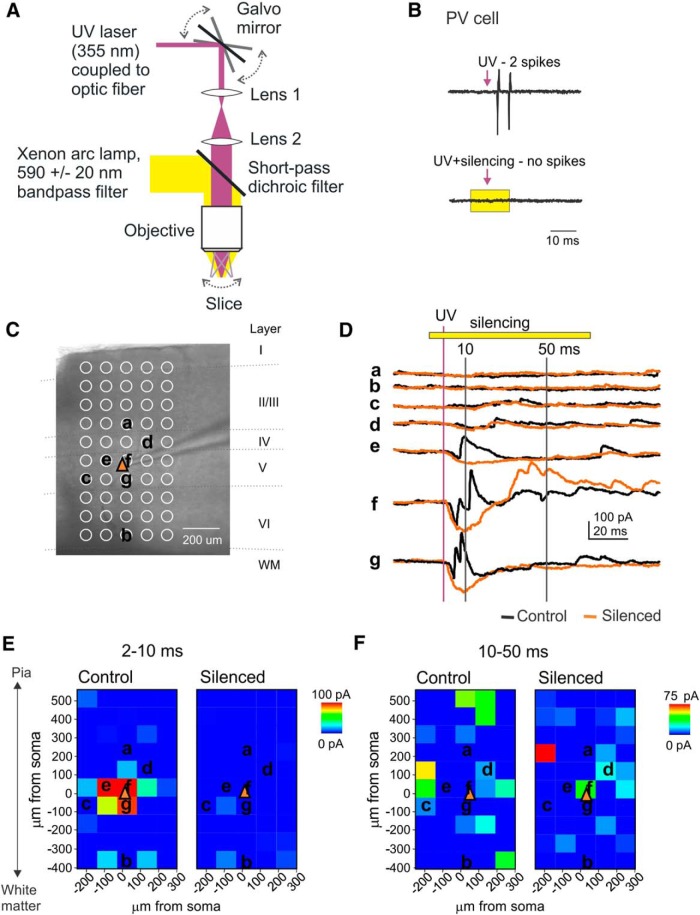
LSPS combined with eNphR-mediated PV cell silencing. ***A***, Diagram depicting setup for LSPS and full-field yellow illumination. The yellow light is reflected by short-pass dichroic filter that transmits UV light. ***B***, Cell-attached recording of an opsin-expressing PV cell that is activated by LSPS only in the absence of eNphR-activating yellow illumination. ***C***, Representative grid for recording LSPS-evoked IPSCs (outward PSCs) in a PYR (orange triangle indicates soma location). Location of cortical layers and white matter is indicated. ***D***, Example traces from the grid in ***C***, at indicated locations. Black traces are without yellow illumination; orange traces are with yellow illumination. Inward responses, largest in perisomatic regions, mainly reflect the direct activation of postsynaptic ionotropic GluRs. The yellow bar indicates illumination. The purple line indicates stimulus onset; vertical lines indicate 10 and 50 ms, the detection times for short- and regular-latency IPSCs. ***E***, Short-latency IPSC maps for the example PYR without (left) and with (right) yellow illumination to silence PV cells. ***F***, Same as ***E***, but for regular-latency ISPCs.

**Figure 4.**
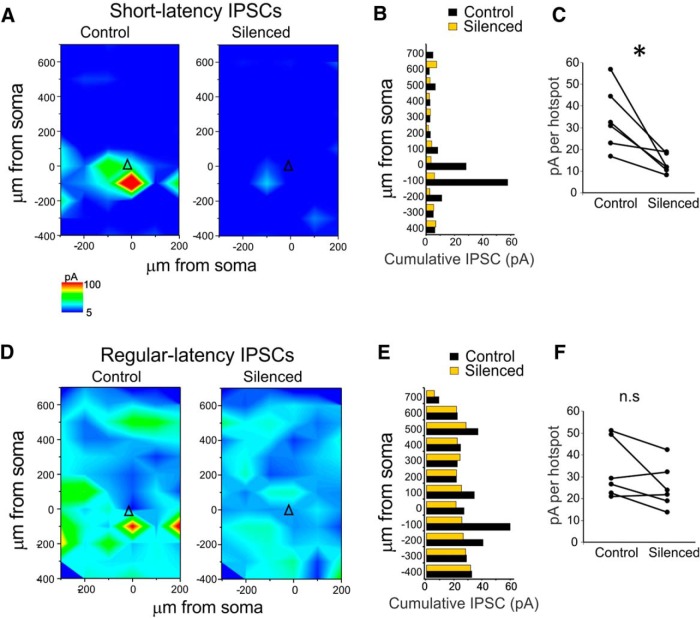
IPSC maps obtained via LSPS with and without PV cell silencing. ***A***, Summary maps for short-latency IPSC maps in PYRs (triangle indicates soma location) recorded without (left) and with (right) yellow illumination. *N* = 6. ***B***, Cumulative IPSC amplitudes (in pA) relative to the horizontal distance from soma without (black) and with (yellow) yellow illumination. ***C***, Cumulative IPSC amplitude per hotspot for all six recorded maps, showing a significant decrease with PV cell silencing. ***D–F***, Same as ***A–C***, but for regular-latency IPSCs. No significant decrease in cumulative amplitude/hotspot with PV cell silencing (***F***). * indicates statistical significance at *p* < 0.05. n.s. = non significant.

Next, we tested whether PV-to-PYR connectivity could be mapped indirectly using a combined approach of optogenetic silencing of eNpHR3.0-expressing PV cells and LSPS. To that end, we directed yellow light (593 nm) through the epifluorescence port of the microscope for full-field illumination via the 63× or 5× objective. The UV light needed for LSPS was directed into the light path prior to the epifluorescence port and transmitted to the objective via a specialized short-pass dichroic mirror (see Materials and Methods). The beam of UV light could be directed to any part of the slice visible through the objective via a pair of Galvo mirrors (Fig. 3*A*). Figure 3*B* shows a cell-attached recording of an eNpHR3.0-expressing layer 5 PV cell (identified by its action potential waveform in cell-attached mode; Brill and Huguenard, 2009), which fires two action potentials in response to LSPS, when the UV light was directed to its soma. In the presence of yellow illumination (15 ms, starting 7 ms before LSPS stimulus), PV cell spiking was abolished (Fig. 3*B*, bottom). Figure 3*C* shows the outline of an LSPS map of IPSCs in a representative pyramidal cell. Maps were recorded twice: once without and once with yellow illumination (−5 to 70 ms relative to UV pulse) to silence PV cells. Yellow illumination led to a substantial reduction in IPSCs recorded in the PYR (Fig. 3*D*, example traces). We separated input maps into IPSCs recorded between 2–10 and 10–50 ms after the LSPS stimulus, to segregate PV- and non-PV cell-mediated inputs, respectively (Brill and Huguenard, 2009). Indeed, in the example cell, short-latency IPSCs (2–10 ms) were almost completely eliminated in the presence of yellow illumination (Fig. 3*E*), while regular-latency inputs (10–40 ms; Fig. 3*F*) were not. This was also true on the population level (*n* = 6 cells from three animals; Fig. 4): short-latency IPSCs were significantly reduced when PV cells were silenced (cumulative IPSCs per hotspot; control, 34.02 ± 5.97 pA; silenced, 13.11 ± 1.76 pA; *p* < 0.05), while regular latency IPSCs remained unchanged (control, 33.23 ± 5.49 pA; silenced, 25.46 ± 4.18 pA; *p* = 0.116). This provides further confirmation that PV-to-PYR connectivity exclusively provides strong, short-latency inhibition.

### Functional role of PV cell-mediated feedforward inhibition

**Figure 5.**
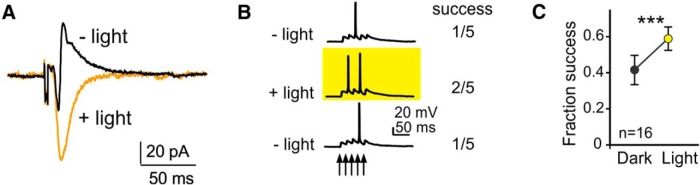
PV cell silencing affects EPSCs in PYRs. ***A***, PSC in a PYR cell in response to electrical stimulation without (black trace) and with (orange trace) PV cell silencing. Note the absence of an IPSC with yellow illumination. ***B***, Response to trains of five electrical stimuli (50 Hz) in a PYR in current clamp without, with, and again without yellow illumination (top to bottom). Note two successes vs. one success of five stimuli with PV cell silencing. ***C***, Quantification of PYR disinhibition due to PV cell silencing, measured as the fraction of successes (action potentials) for five-pulse stimuli, as shown in ***B***.

Having established that LSPS-activated PV-to-PYR connections can be efficiently silenced by eNpHR3.0-mediated hyperpolarization of PV cells, we wanted to determine how feedforward EPSCs in PYRs are affected by PV cell silencing, which would likely increase PYR excitation and reveal occult connections. In a series of preliminary experiments, we used electrical stimulation in layer 2/3 to determine whether yellow illumination of eNpHR3.0-expressing PV cells could alter functional output of PYRs in layer 5. When recorded at a holding potential of −40 mV electrical stimuli typically resulted in biphasic responses consisting of an early EPSC followed by an IPSC, the latter of which was abolished by yellow illumination (Fig. 5*A*). When giving trains of five electrical stimuli at 50 Hz and recording in current-clamp, yellow illumination (i.e., silencing of PV cells) increased action potential generation: while the success rate (fraction of stimuli resulting in spikes) was 0.41 ± 0.08 in control conditions, it was increased to 0.59 ± 0.06 with PV silencing (*p* < 0.001; *n* = 16 cells from eight animals; Fig. 5*B*,*C*). Thus, we show that PV cell silencing can significantly impact functional PYR output.

**Figure 6.**
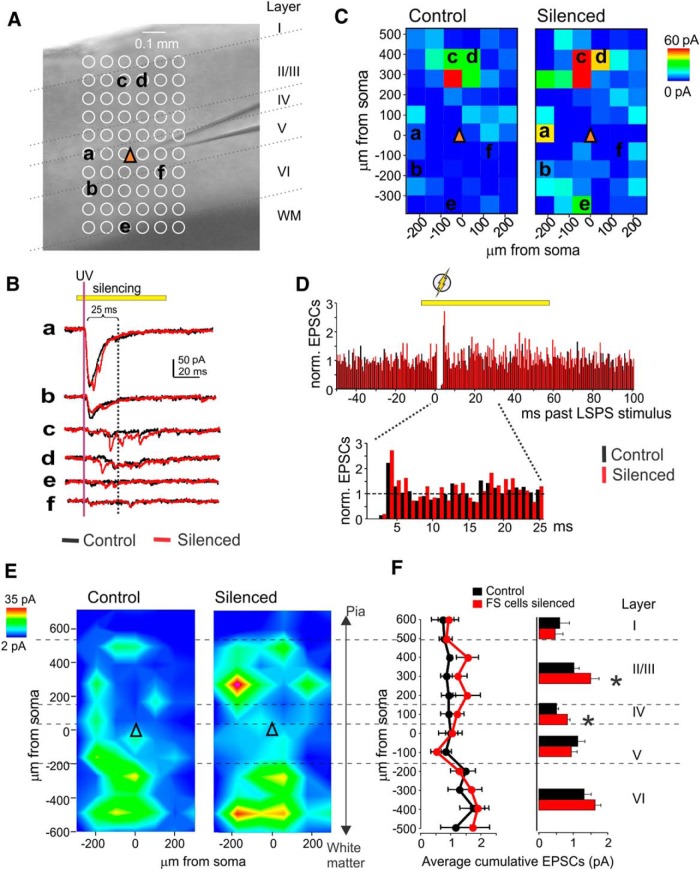
PV cell silencing unmasks excitatory connectivity. ***A***, Representative grid for recording LSPS-evoked EPSCs in a PYR (yellow triangle indicates soma location). Location of cortical layers and white matter is indicated. ***B***, Example traces from the grid in ***A***, at indicated locations. Black traces are without yellow illumination, and red traces are with yellow illumination. Yellow bar indicates yellow illumination; purple line indicates UV stimulus; black line indicates 25 ms post-UV stimulus (end of EPSC detection window). ***C***, EPSC maps for the example PYR without (left) and with (right) yellow illumination to silence PV cells. ***D***, Peristimulus time histogram for EPSCs recorded in 11 pyramidal cells. All detected EPSCs (spontaneous and LSPS evoked) during all sweeps from all 11 cells were sorted into 1 ms bins relative to LSPS stimulus and normalized to the average EPSC rate in controls (100 ms preceding LSPS stimulus). Black, Control (no yellow illumination); red, with PV cell silencing. EPSC rates are elevated following LSPS stimulus but return to baseline within ∼10 ms, indicating that evoked EPSCs are predominantly monosynaptic. Bottom, Zoomed-in view of the 25 ms following LSPS stimulus, which were used as a detection window for evoked EPSCs. ***E***, Summary EPSC maps in PYRs recorded without (left) and with (right) yellow illumination. *N* = 11. ***F***, Cumulative EPSC amplitudes relative to the horizontal distance from soma in 100 μm bins (left) and for each neocortical layer (right). * indicates statistical significance at *p* < 0.05.

Next, we used the combined LSPS/optogenetics approach outlined above, but instead of IPSCs, we recorded EPSCs by holding PYRs at −60 mV, very close to the chloride equilibrium potential. LSPS excitatory synaptic input maps were recorded under control conditions and in the presence of full-field yellow illumination (continually from 7 ms before until 60 ms after LSPS stimulus), and an EPSC detection window of 3–25 ms post-LSPS stimulus was used. Figure 6*A–C* shows example traces from a map recorded in a representative layer 5 PYR with and without yellow illumination, and the resulting EPSC maps. Silencing of eNpHR3.0-expressing PV cells lead to an increase in excitatory inputs (Fig. 6*B*,*C*, example traces c and d). A peristimulus time histogram (Fig. 6*D*) shows the average number of EPSCs recorded during all sweeps in the 11 cells, normalized to control (no illumination) values. EPSC rates were transiently elevated following LSPS stimulus, but returned to baseline values within 10 ms. This indicates that most of the detected LSPS-evoked EPSCs are monosynaptic. On the population level (*n* = 11 cells from seven animals), silencing PV cells resulted in significant increases in LSPS-mediated EPSC input onto layer 5 PYRs, especially from layers 2/3 (average cumulative EPSC per spot: control, 1.06 ± 0.15 pA; silenced, 1.51 ± 0.25 pA; *p* < 0.05) and layer 4 (control, 0.51 ± 0.07; silenced, 0.84 ± 0.08; *p* < 0.001; Fig. 6*E*,*F*). We conclude that PV cell silencing can unmask excitatory monosynaptic inputs onto PYRs in a layer-specific manner.

## Discussion

In this study, we outline two methods for obtaining cell type-specific synaptic input maps: first, using focal optogenetic activation of target cells (PV cells in this case) to map PV-to-PYR connections; and second, a combined approach of LSPS- and eNpHR3.0-mediated silencing of PV cells to observe direct and indirect effects on inhibitory and excitatory connectivity, respectively.

Using targeted focal activation of ChETA-expressing PV cells, we mapped PV-to-PYR inhibitory inputs. We previously showed that LSPS-evoked inhibitory inputs onto PYRs could be roughly subdivided into the following two classes (short-latency, high-amplitude inputs; and longer-latency, lower-amplitude inputs), and we hypothesized that those were mediated by PV and non-PV cells, respectively (Brill and Huguenard, 2009). Results from the ChETA-based mapping approach support this idea, as the location and latency of PV-to-PYR inputs were very similar to short-latency ISPCs. Our results are also consistent with connectivity maps acquired using two-photon LSPS to excite individual GFP-tagged PV cells (Packer and Yuste, 2011). We also noted essentially no long-distance short-latency inputs, indicating that there are no prominent long-range PV cell-mediated connections to layer 5 (unlike long-range connections from layer 5 to layer 2/3; Buchanan et al., 2012).

In a second set of experiments, we activated cells by conventional LSPS either with or without silencing of eNpHR3.0-expressing PV cells. In this approach, rather than directly measuring PV-to-PYR inputs, we identified them indirectly by deducing their influence on the overall map. Again, we were able to conclude that PV cell-mediated inputs onto PYRs were of the short-latency variety and originated from the area surrounding the somata of the recorded PYRs. Since this indirect approach used LSPS for cell activation, it may have been a more direct comparison to the original study (Brill and Huguenard, 2009) than the ChETA-based input mapping. This circumvents potential confounders such as the ability of channelrhodopsins to activate axons, which LSPS does not (see above). It is of interest to note that the LSPS study that identified short-latency versus regular-latency inputs used neocortical slices from rats (Brill and Huguenard, 2009), while the present study was done using mice. We conclude that the focal and dynamic profiles of PV-to-PYR connectivity in both species of rodents are quite similar. Since not all PV cells were infected with opsins (Fig. 1), residual short-latency IPSCs might stem from uninfected (non-eNpHR3.0-expressing) PV cells.

Both of these approaches (direct ChETA-based input mapping and LSPS/eNpHR3.0-based mapping) can be used to construct input maps between any two cell types, thus facilitating medium-/high-throughput, cell type-specific quantitative connectivity mapping. Using a soma- or axon initial segment-targeting channelrhodopsin variant (Grubb and Burrone, 2010) would enhance the spatial specificity of the direct approach. We should also caution that a response to glutamate uncaging or optogenetic stimulation indicates the presence of a presynaptic cell, but that the lack of a response does not indicate the lack of a presynaptic cell. Our methods thus make a statistical argument for connectivity patterns, but do not identify connections exhaustively.

We also recorded excitatory LSPS-evoked inputs in the presence and absence of eNpHR3.0-mediated PV cell silencing. We show that PV cell silencing unmasks significant, layer-specific EPSC inputs. Leading into this set of experiments, we also demonstrate that PV cell silencing increases the spike output of PYRs when subjected to a train of electrical stimuli. We assume that the mechanism that accounts for this effect is the following: electrical stimulation activates not only axons of excitatory cells that target the recorded PYRs, but also those of local interneurons. Under control conditions, suprathreshold and rapid activation of PV cells in proximity of the PYRs results in a recurrent inhibition in those PYRs that is powerful enough to suppress their direct excitation and action potential output by uncaged glutamate. When this inhibition is silenced, PYR spike output increases.

Focal LSPS with PV cell silencing likely relies on PV-to-PYR feedforward inhibition, too. For excitatory inputs to be received by the recorded PYR (located elsewhere), PV-to-PYR feedforward inhibition must be overcome at the LSPS stimulation site. When subjected to LSPS of equal intensity, PV cells have significantly shorter spike latencies than PYRs (Brill and Huguenard, 2009); thus, it is plausible that the simultaneous activation of PYRs and PV cells by LSPS still results in significant functional feedforward inhibition of PYRs. Notably, the strength of this feedforward inhibition appears to vary between cortical layers, as it is especially prominent in upper layers.

Our results show that PV cell activity can effectively occlude excitatory connectivity between PYRs. In other words, the silencing of PV cells reveals many occult connections that were masked by powerful feedforward inhibition. This is especially relevant for studies comparing excitatory connectivity during development or in disease models. For example, epilepsy/hyperexcitability is frequently characterized by increased excitatory connectivity as a result of axonal sprouting (for review, see Marco and deFelipe, 1997; Jin et al., 2006; Chu et al., 2010; Zhang et al., 2012; Buckmaster, 2014; Kúsmierczak et al., 2015), but changes in inhibitory connectivity have also been reported (Brill and Huguenard, 2010; Zhou and Roper, 2010; Jin et al., 2011, 2014). When quantifying excitatory connectivity changes, one should bear in mind that decreased interneuron excitability or interneuron loss might enhance net excitatory inputs due to a lack of feedforward inhibition, and vice versa. The ultimate “clean” experiment would be to record EPSC input in the presence of GABAergic blockers; but, unfortunately, this will rarely be possible due to the emergence of epileptiform activity in fully disinhibited slices (Chagnac-Amitai and Connors, 1989). Brief disinhibition of only a subset of interneurons, as described here, can circumvent this problem, as we did not observe epileptiform activity during our experiments.

Using opsin-expressing interneuron subtypes as a tool for synaptic connectivity mapping alone or in combination with LSPS is a promising way to obtain quantitative information on interneuron connectivity patterns. It can also shed light on phenomena such as feedforward inhibition and its impact on connectivity mapping.

## Synthesis

The decision was a result of the Reviewing Editor Christian Hansel and the peer reviewers coming together and discussing their recommendations until a consensus was reached. A fact-based synthesis statement explaining their decision and outlining what is needed to prepare a revision is listed below. The following reviewers agreed to reveal their identity: Andreas Frick, David McCormick

This is a techniques manuscript, which describes an interesting way to map inputs to cortical neurons using optogenetics. They authors use optogenetics and local light application in slices to map inhibitory connections to pyramidal cells and how altering inhibition controls the flow of excitation in the local network. Although not entirely novel, this approach is of interest, provided that the manuscript is appropriately revised.

Major comments:

- The authors find that PV-mediated inhibition is restricted to the proximal region of layer 5 pyramidal neurons. This is based on electrophysiological recordings performed at the cell bodies of these neurons. While the majority of PV cells might target the axo-somatic or proximal dendritic region of pyramidal neurons, some might also target more distal dendrites. For example, Buchanan et al (2012, Neuron) describe a fairly common layer 5 Pv+ interneuron with an ascending axonal arbor, mediating inhibition within layer 2/3. It is possible that this interneuron type also provides direct dendritic inhibition of layer 5 pyramidal neurons in these supra-granular layers. Inhibition targeting distal dendrites (either in the form of IPSCs or as shunting conductances) might therefore easily be missed with somatic recordings. Please discuss.

- The potential shortcomings of both methods (glutamate uncaging and ChETA activation) in the context of their results should be discussed in more detail. As for glutamate uncaging, the authors state that this is a suitable method to effectively map the somatic location of neurons providing input to a recorded neuron. A lack in response following glutamate stimulation, however, may be due to either a lack in connectivity or the connections being severed. As such, only the small subset of local projections that are presented in a slice are being probed.

- ChETA activation. Because Cheta-positive axons can be photo-stimulated even if they are severed from their parent cell body, this method can be used to map circuits that are not preserved in brain slices. Despite this obvious advantage, release from excited axons may pose other problems regarding the interpretation of the obtained results. For example, stimulation of axons within the infragranular layers of supragranular PV+ interneurons may confound the interpretation of which layer the inhibition is coming from.

- The time window (up to 50 ms) for onset latencies of IPSCs used in this study to quantify responses seems rather long. PV+ interneurons are known to have extremely fast signaling properties, leading to fast speeds of feedforward and feedback inhibition - for example, the latency of disynaptic inhibition under physiological conditions is only 2 ms or less (reviewed by Hu et al., 2014, Science). That means that within a time window of 50 ms, the authors will measure a mixed bag of various forms of inhibition. To reduce polysynaptic activation, they use NMDA receptor blockers, but they should demonstrate the efficacy of this approach. For example, blockers of NMDA receptors may reduce release onto certain types of interneurons, but not others (e.g. Buchanan et al, 2012, Neuron).

- Latency of IPSCs. Some of the IPSCs have much longer latencies than others (e.g. compare traces b and c in Fig. 3B), which are unlikely the result of different travel times required to reach the cell body of the pyramidal neuron. As stated above, PV+ interneurons are known to have extremely fast signaling properties. What could be the explanation for the very different latencies? Other responses show that multiple IPSCs sum up in a facilitating manner (e.g. trace d in the same Figure). Have the authors tested whether the short stimuli (few ms) used for ChETA activation can evoke a train of APs in Pv+ interneurons? If this was the case, transmitter release failures in response to the first APs could also explain the long latencies of some of these responses.

- Experiments investigating the functional role of PV-cell silencing on feed-forward EPSCs in PYRs (Figure 6). How can the authors tease apart the contribution of feedforward and feedback inhibition impinging on layer 2/3 PYRs and layer 5 PYRs, respectively?

Minor comments:

- What is the age of the mice used?

- Was the co-expression of parvalbumin and YFP quantified for both ChETA and eNpHR constructs? Is the eYFP expression in Fig. 1 associated with ChETA or eNpHR expression?
